# A Systematic Review and Meta-Analysis of Systemic Antibiotic Therapy in the Treatment of Peri-Implantitis

**DOI:** 10.3390/ijerph19116502

**Published:** 2022-05-26

**Authors:** Manuel Toledano-Osorio, Cristina Vallecillo, Raquel Toledano, Fátima S. Aguilera, María T. Osorio, Esther Muñoz-Soto, Franklin García-Godoy, Marta Vallecillo-Rivas

**Affiliations:** 1 Department of Stomatology, Faculty of Dentistry, University of Granada, Colegio Máximo de Cartuja s/n, 18071 Granada, Spain; mtoledano@ugr.es (M.T.-O.); fatimas@ugr.es (F.S.A.); emsoto@ugr.es (E.M.-S.); mvallecillo@correo.ugr.es (M.V.-R.); 2 Independent Researcher, 18071 Granada, Spain; rtoleosorio@gmail.com (R.T.); mtoleosorio@gmail.com (M.T.O.); 3 Health Science Center, College of Dentistry, University of Tennessee, 875 Union Avenue, Memphis, TN 38103, USA; fgarciagodoy@gmail.com

**Keywords:** antibiotics, bleeding on probing, peri-implantitis, probing pocket depth, antibiotic resistance, antibacterial agents

## Abstract

Research has been conducted into the advantages of the systemic administration of antibiotics. The aim of this systematic review and meta-analysis was to assess the efficacy of systemic antibiotic administration in the treatment of peri-implantitis in terms of bleeding on probing (BoP) and probing pocket depth (PPD). Literature searches were performed across PubMed, EMBASE, and Cochrane Central Register of Controlled Trials (CENTRAL) to identify randomized controlled trials and observational clinical studies. After peri-implantitis treatment, PPD was reduced by 0.1 mm (*p* = 0.58; IC 95% [−0.24, 0.47]), indicating a non-significant effect of antibiotic administration on PPD. The BoP odds ratio value was 1.15 (*p* = 0.5; IC 95% [0.75, 1.75]), indicating that the likelihood of bleeding is almost similar between the test and control groups. Secondary outcomes were found, such as reduced clinical attachment level, lower suppuration and recession, less bone loss, and a reduction in total bacterial counts. In the treatment of peri-implantitis, the systemic antibiotic application reduces neither PPD nor BoP. Therefore, the systemic administration of antibiotics, in the case of peri-implantitis, should be rethought in light of the present results, contributing to address the problem of increasing antibiotic resistance.

## 1. Introduction

Dental implant therapy is one of the most common treatments for replacing missing teeth [1]. Peri-implantitis is a common biological complication in patients with implant-supported prosthesis [2]. According to the 2017 World Workshop on the Classification of Periodontal and Peri-Implant Diseases and Conditions (2017 WWP), the definition of peri-implantitis includes (1) bleeding and/or suppuration on gentle probing, (2) increased probing pocket depth compared to previous examinations and (3) bone loss. This definition requires the existence of a previous examination of the patient, which is not always available. If no previous registers are available, alternative diagnostic criteria have been proposed: (1) bleeding and/or suppuration on gentle probing, (2) probing pocket depths of ≥6 mm and (3) bone levels ≥3 mm apical of the most coronal portion of the intra-osseous part of the implant [3].

In the pathogenesis of peri-implantitis, microbial colonization of the implant surface is the main causative factor [4]. Peri-implant biofilm contains a complex array of bacterial species that trigger the infection and cause the beginning of the disease [5]. The microbiota in peri-implantitis seems to be mainly composed of anaerobic Gram-negative species and is not linked to a uniform microbial profile, contrary to periodontitis [6]. A marked difference has not been detected in the bacterial species between periodontal and peri-implant sites [7,8]. However, some species have shown higher counts in peri-implantitis, particularly: *Tannerella forsythia, Treponema denticola, Campylobacter rectus, Treponema socranskii, Porphyromonas gingivalis, Staphylococcus aureus, Campylobacter gracilis* and *Prevotella intermedia* [7,8,9]. This cluster of bacteria has been associated with the onset of peri-implantitis [10,11]. In normal peri-implant health, these bacterial communities are in equilibrium with the host; however, the presence of some risk factors that favor biofilm formation could trigger the alteration of the microenvironment and tissue inflammation. This condition, in conjunction with poor oral hygiene, could increase the counts of these species, as previously described [12]. The development of a complex infectious microbiota represents a clinical challenge in peri-implantitis management [13]. The non-linear accelerative progressive pattern of bone loss in peri-implantitis leads to implant failure if the given infection is not proficiently arrested [14]. Thereby, the treatment of peri-implantitis aims to control the infection and reduce bacterial load.

In order to achieve this purpose, clinicians attempt to successfully treat peri-implantitis with non-surgical approaches (i.e., mechanical debridement) often in association with adjuncts (local and systemic antimicrobials, lasers, photodynamic therapy, etc.). Adjunctive use of antibiotics within the treatment of peri-implantitis can be performed locally or systemically. In a recent systematic review and meta-analysis, the local application of antibiotics was demonstrated to have benefits when treating peri-implantitis without the occurrence of any adverse effect [15]. However, the topical application of antibiotics, requires in many cases, the exposure of the implant surface and the bone defect. Some case series and cohort studies showed added benefit to non-surgical therapy when systemic antibiotics were used adjunctively [16,17,18]. Significant radiographic defect fill has also been reported after prescribing systemic antibiotics as an adjunctive to non-surgical therapy [19]. Most surgical treatment protocols for peri-implantitis suggest the adjunctive use of systemic antibiotics to reduce the counts of specific putative bacteria [20,21]. It could, therefore, be argued that antibiotics may be necessary to resolve the infection [22].

The delivery and route of the drug through the blood to the target organ is one of the main mechanisms of systemic antimicrobials. In addition, the systemic administration of antibiotics allows for greater bioavailability in oral tissues, resulting in oral disinfection regardless of the location of the peri-implant pocket [23]. Based upon the current understanding that peri-implantitis and periodontitis share a similar infectious etiology, the use of systemic antibiotics for the therapy of peri-implantitis has been advocated [24]. Thereby, systemic antimicrobial administration is considered by some authors the standard in the management of peri-implant diseases [1]. Nevertheless, some concerns have arisen over the generalized use of antibiotics. There has been a spectacular and rapid evolution of antibiotic-resistant strains of bacteria, associated with the indiscriminate usage of antibiotics, over the last 60 years [25]. This has culminated in the appearance of pathogens with resistance to a wide range of antibiotics, and a rise in similarly resistant opportunistic pathogens. Antibiotic resistance is a critical and growing problem for humans, and is recognized as such by governments, clinical practice, research, and industry [26].

Microbiological diagnosis was proposed as a possible approach to detect the most aggressive periodontal pathogens [27]. Since in some patients there is not a marked difference in the bacterial species between periodontal/peri-implant health and disease, these microbiological culture tests are reserved only for those cases of disease in which there is not a good response to basic therapy [27,28].

Consequently, the main goals of peri-implantitis treatment must be the resolution of soft tissue inflammation (i.e., absence of bleeding and suppuration) and the maintenance/stability of supporting tissues (absence of bone loss) [29]. Research has been conducted into the advantages of the systemic administration of antibiotics, which may allow high concentrations to be maintained in the peri-implant bone defect, potentially causing a reduction in both probing pocket depth (PPD) and bleeding on probing (BoP) [30,31]. The aim of this systematic review was, therefore, to address the following focused question: In patients requiring peri-implantitis treatment, what efficacy of systemic antibiotic administration, in terms of PPD and BoP reduction, could be expected?

## 2. Materials and Methods

### 2.1. Protocol and Registration

The study protocol was prepared in consideration of the Preferred Reporting Items for Systematic Review and Meta-Analysis (PRISMA) statement and increasing the transparency of the review using the PRISMA checklist [32]. The developed protocol was previously registered and allocated the identification number CRD42021267959 in the International Prospective Register of Systematic Reviews (PROSPERO) database, hosted by the National Institute for Health Research, University of York, Center for Reviews and Dissemination (www.crd.york.ac.uk/PROSPERO (accessed on 25 May 2022)).

### 2.2. Focused Question

The focused query was designed according to the PICO question [33]: In patients requiring peri-implantitis treatment, what efficacy of systemic antibiotic administration, in terms of probing pocket depth and bleeding on probing reduction, could be expected more than 3 months postoperatively?

The PICOs elements were as follows:

Population (P): Patients with peri-implantitis.
Intervention (I): Peri-implantitis treatment performed with systemic antibiotic therapy with pre- and post-operative clinical evaluation.
Comparison (C): Peri-implantitis treatment performed without systemic antibiotic adjunctive therapy with pre- and post-operative clinical evaluation.
Outcome (O): Outcomes measuring changes in clinical parameters including PPD and BoP, at implant, before and after (at least 3 months) peri-implantitis treatment.
Study (S): Randomized controlled trials (RCTs) and observational studies (cohort and case–control studies and case series).

### 2.3. Search Strategy

Peer-reviewed publications up to July 2021 across PubMed, EMBASE and Cochrane Central Register of Controlled Trials (CENTRAL) were searched for eligibility. Only studies published in English between 1992 and July 2021 were considered. Reference lists of the previous reviews and included studies were screened to search for relevant manuscripts that were missing after the electronic screening. Bibliographies of eligible articles were manually searched.

The following electronic database search keywords were used: (periimplantitis OR “peri-implantitis” OR “peri-implant infection” OR “peri-implant disease” OR “peri-implant bone loss” OR “periimplant mucositis” OR “peri-implant mucositis” OR “periimplant” OR “peri-implant” OR “dental implant inflammation”) AND ((antibiotics or “antibiotic” or “antimicrobial” or “anti-microbial” or “anti-infective agents” or “bactericides”) AND (“systemic”)).

### 2.4. Eligibility: Inclusion and Exclusion Criteria for Studies

An article was included if it involved the following:

1. For clinical studies, publications of adult subjects in good general health and at least a three-month follow-up period.
2. Studies performing an explicit diagnosis of peri-implantitis.
3. Studies assessing the effectiveness by comparing changes in clinical parameters including PPD reduction and BoP reduction.

The exclusion criteria:

- In vitro and pre-clinical studies, systematic reviews.
- Full-text publications not available in the English language.
- Studies with less than 3 months of follow-up.

### 2.5. Study Selection and Data Extraction

Electronic and manual literature searches were conducted by 2 independent reviewers (M.T.-O. and C.V.), who selected eligible studies by reviewing the list of titles and abstracts and considering the inclusion and exclusion criteria. The complete articles sourced via eligible titles and abstracts were obtained and examined independently to determine eligibility. Disagreements between these reviewers related to the selection and inclusion of any specific paper were discussed until either a consensus was reached, or a third reviewer (M.V.-R.) facilitated an agreement and determined inclusion or exclusion. In order to measure the agreement between the two reviewers, Cohen’s Kappa-coefficient was calculated. All reports excluded at this stage were formally recorded, as well as the reason/s for their exclusion.

Two investigators (M.T.-O. and C.V.) independently extracted the data from included articles and assessed the risk of bias in duplicate, and thereafter discussed their conclusions to find an agreement. In case of disagreement, the judgment of a third reviewer (M.V.-R.) was decisive. Data extracted were: (1) authors and year of publication; (2) study design; (3) number of patients and implants; (4) peri-implantitis treatment; (5) antibiotic and dosage; (6) follow-up time; (7) BoP reduction; and (8) PPD reduction.

Additionally, data concerning sample size, age of participants, PI clinical criterion, number of sites measured per implant, microbiological evaluation, biomarker measurement in gingival fluid, systemic or radiological outcomes and adverse effects were also registered. To complete the search, information regarding secondary outcomes (plaque score, gingival index, clinical attachment level, suppuration, recession, keratinized mucosa, bone loss, total bacterial counts and adverse events) was also reported.

### 2.6. Assessment of Risk of Bias

Methodological quality and risk of bias were evaluated according to: (i) The Cochrane Collaboration’s tool [34]. After analyzing different domains of bias, studies were classified as “high risk”, “some concerns” or “low risk”; (ii) The Joanna Briggs Institute Critical Appraisal tool for the included non-randomized studies. Reviewers independently scored the papers and considered as having a high, medium or low risk of bias [35].

### 2.7. Data Analyses

For the primary outcomes, PPD reduction [in terms of PPD reduction (mm)] and BoP reduction (in terms of percentage of implants with bleeding on probing reduction), descriptive statistics were used. For PPD reduction, weighted means (CI 95%) were calculated, including total sample size, inverse variance and standard error of the treatment effect. For BoP reduction, the odds ratio (OR) (CI 95%) was calculated using the chi-square test [Mantel–Haenszel (M–H)]. The variation across the included studies, or heterogeneity, was determined using Higgins (*I^2^*). Random-effects models were applied in order to analyze effect sizes. Three subgroups within each primary outcome (PPD and BoP) were established. Hence, comparative studies between experimental and control groups considering the (i) time of follow-up (≤3 months, >3 months), (ii) application of surgical therapy or not, and (iii) the most commonly cited types of antibiotics administered (azithromycin (AZM), amoxicillin plus metronidazole (AMX + MTZ) and AMX) were also performed for both primary outcomes. Data were analyzed with RevMan 5.4 (The Cochrane Collaboration, Oxford, UK). A funnel plot was also produced by RevMan 5.4 (The Cochrane Collaboration, Oxford, UK) to represent systematic heterogeneity. Statistical significance was set at 0.05.

## 3. Results

### 3.1. Search Results

**Figure 1:**
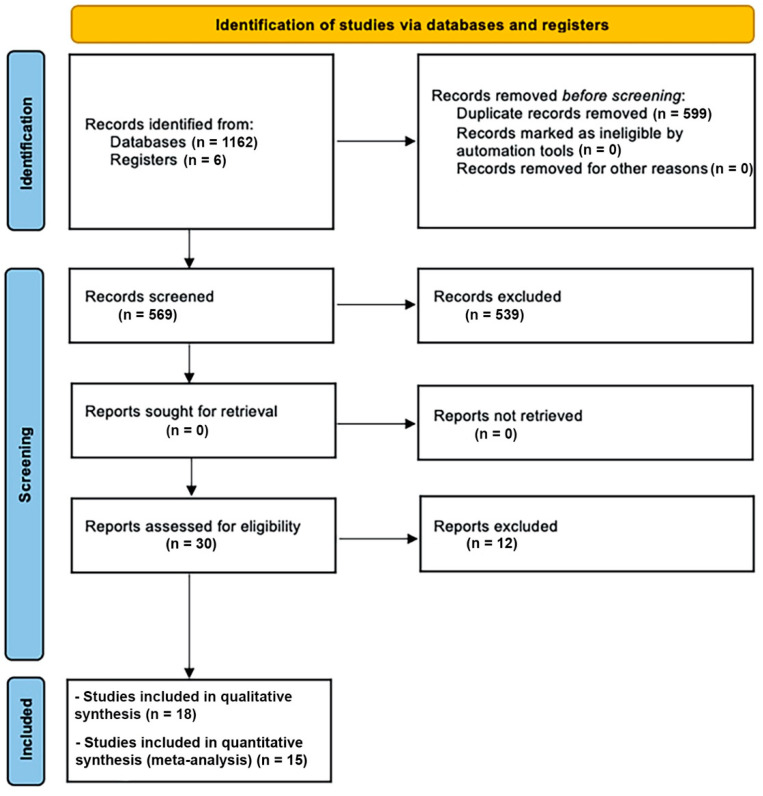
PRISMA flow diagram for the studies inclusion process.

Throughout the electronic search performed in July 2021, 1162 articles with potentially eligible records were found. Manual search was used to identify six more manuscripts. Subsequent to duplicate removal and after the reading of titles and/or abstracts, 30 articles were selected. Then, the full-text version of all the selected articles was reviewed for the inclusion criteria. Following the evaluation and deep reading of articles, 12 were excluded (Table 1). Therefore, 18 articles were included in the final selection and reserved for data extraction. Of the 18 included articles, nine were RCTs and nine non-RCTs (prospective studies). The Cohen’s Kappa results during the selection of the included studies were 0.91 and 0.89. A flowchart of the selection and inclusion method undertaken in the meta-analysis process, based on PRISMA recommendations, is presented in Figure 1. The extracted data for each reviewed article are shown in Table 2.

### 3.2. Studies Quality Assessment and Bias Risk

**Figure 2:**
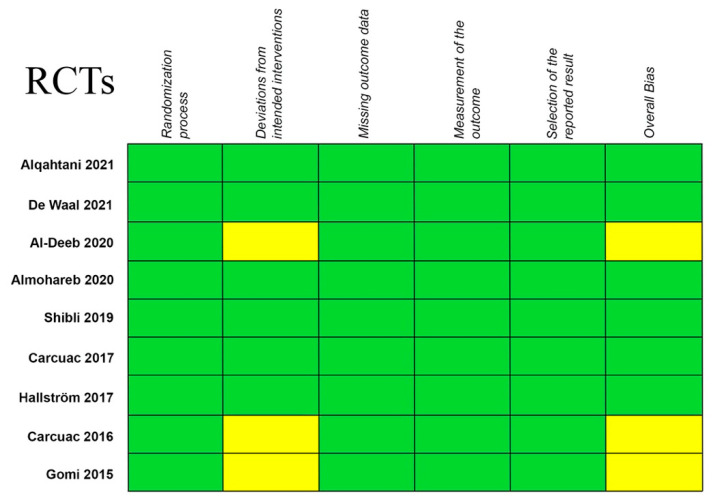
Quality evaluation of the RCTs using the Robins-II Tool. The risk of bias of the included studies was judged as low (green), some concerns (yellow) or high (red) [1,6,24,30,48,51,54,55,56].

**Figure 3:**
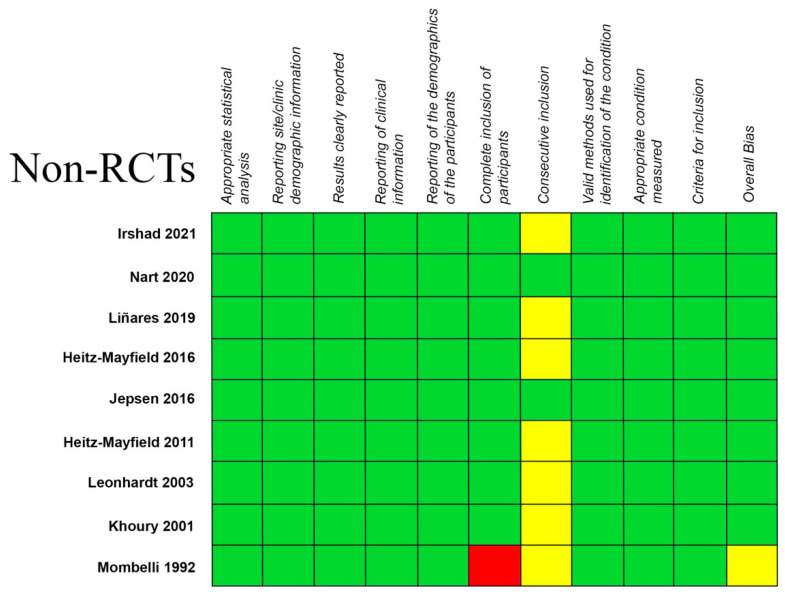
Quality evaluation of the non-RCTs using The Joanna Briggs Institute Critical Appraisal tool. The risk of bias of the included studies was contemplated as low (green), unclear (yellow) or high (red) [16,17,22,47,49,50,52,53,57].

The results in terms of quality assessment and the bias risk of the selected studies are summarized in Figure 2 and Figure 3. Most of the selected papers were considered as having a low risk of bias.

### 3.3. Primary and Secondary Outcomes

Eighteen studies (605 patients and 870 implants) examined both the PPD reduction and BoP reduction. The general characteristics of the included studies are displayed in Table 2.

**Figure 4:**
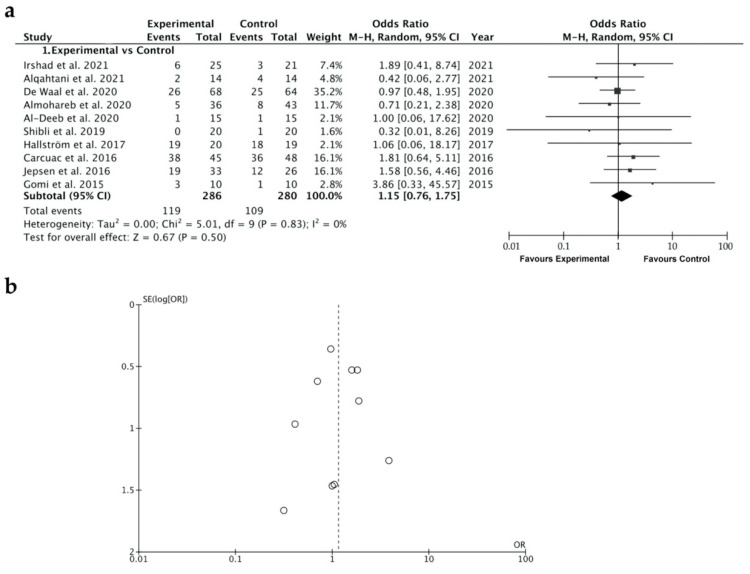
(**a**) Forest plot for no systemic antibiotic (control group) versus adjunct systemic antibiotic (test group) when comparing the bleeding on probing (BoP). (**b**) Funnel plot graph illustrating the publication bias and the systematic heterogeneity of the included studies. The standard error (SE) is represented in the vertical axis and the bleeding on probing (MD) in the horizontal axis [1,6,24,30,48,52,54,55,56,57].

**Figure 5:**
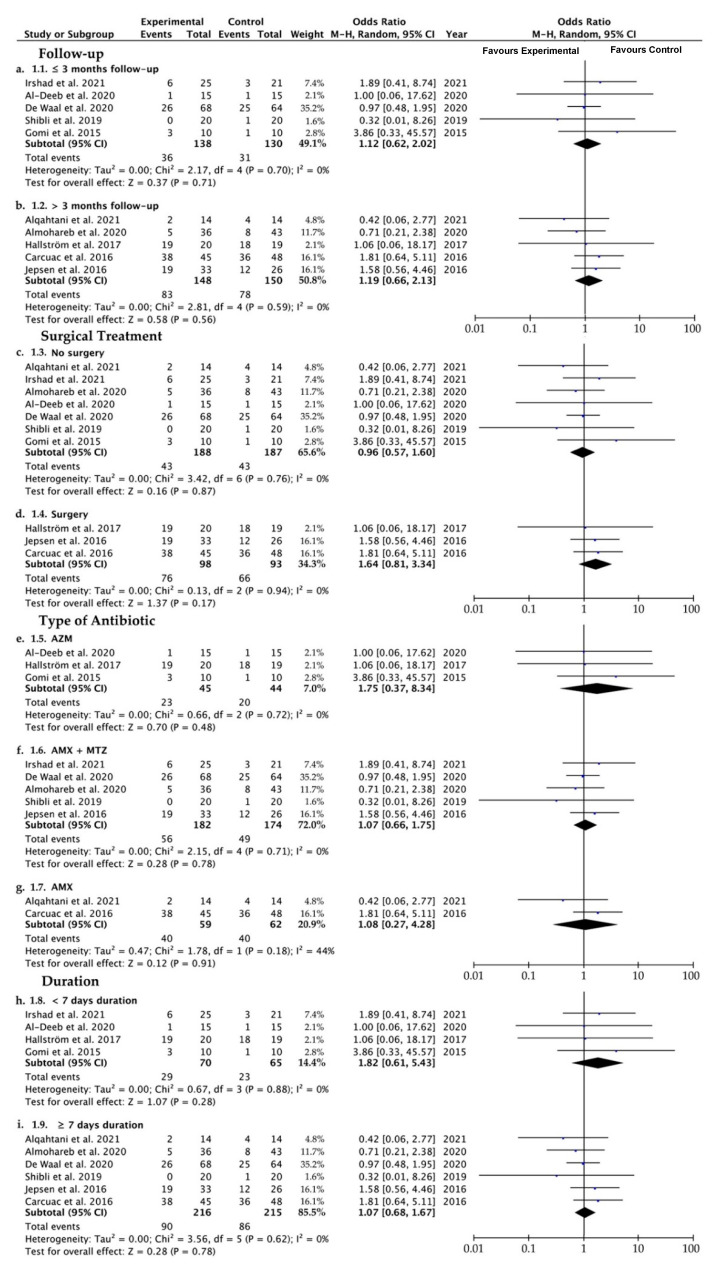
Forest plot for no systemic antibiotic (control group) versus adjunct systemic antibiotic (test group) when comparing the bleeding on probing (BoP) after (**a**) three months or less of follow-up, (**b**) more than 3 months of follow-up, (**c**) performing an implant exposure surgery or open flat debridement, (**d**) performing non-surgical subgingival debridement, (**e**) azithromycin as adjunct systemic antibiotic, (**f**) metronidazole plus amoxicillin as adjunct systemic antibiotic, (**g**) amoxicillin as adjunct systemic antibiotic, (**h**) less of 7 days of antibiotic administration, and (**i**) more than 7 days of antibiotic administration. Weighted mean is presented at CI 95%. Heterogeneity was determined using Higgins (I^2^). In all the analyses, a random-effects model was applied. Statistical significance was set at 0.05 [1,6,24,30,48,52,54,55,56,57].

The reduction in the OR of BoP, when comparing experimental and control groups, was 1.15, ranging from 0.76 to 1.75 (CI 95%) (*p* = 0.5), suggesting that the likelihood of bleeding is similar when antibiotics are systemically administered or not. Heterogeneity was not found (I^2^ = 0%) and the significance of the random-effect model was *p* = 0.50 (Figure 4a). The BoP forest plot graph is given in Figure 4b. Systematic heterogeneity is reflected in the funnel plot graph (Figure 4b). The comparative studies performed in the four subgroups (time of follow-up, surgical therapy or not, type of antibiotics, and duration of antibiotic regimen) did not show significant differences when the control and the test groups, considering BoP, were analyzed (Figure 5).

**Figure 6:**
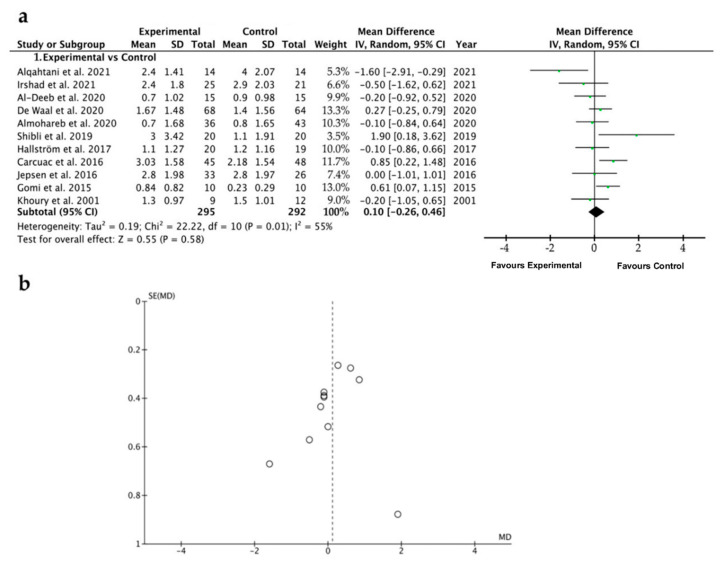
(**a**) Forest plot for no systemic antibiotic (control group) versus adjunct systemic antibiotic (test group) when comparing peri-implant probing pocket depth (PPD). (**b**) Funnel plot graph illustrating the publication bias and the systematic heterogeneity of the included studies. The standard error (SE) is represented in the vertical axis and the probing pocket depth (MD) in the horizontal axis [1,6,17,24,30,48,52,54,55,56,57].

**Figure 7:**
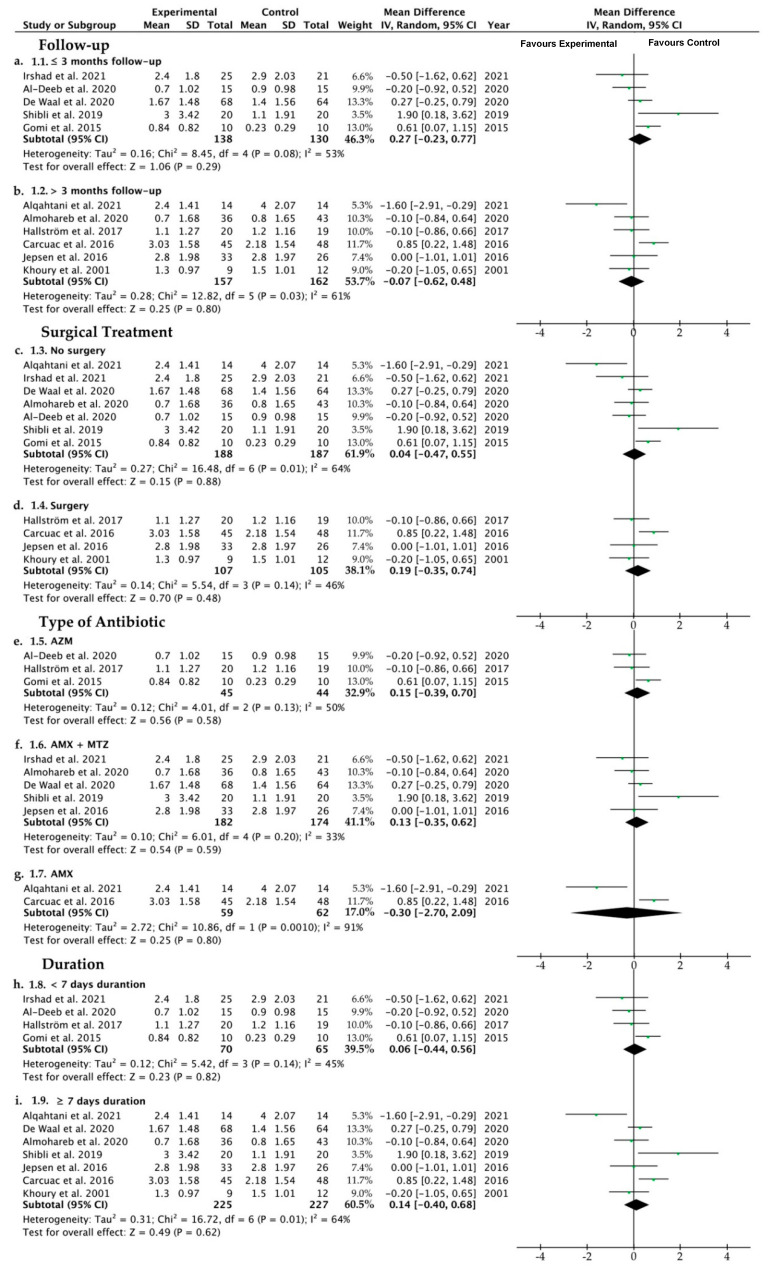
Forest plot for no systemic antibiotic (control group) versus adjunct systemic antibiotic (test group) when comparing peri-implant probing pocket depth (PPD) after (**a**) three months or less of follow-up, (**b**) more than 3 months of follow-up, (**c**) performing an implant exposure surgery or open flat debridement, (**d**) performing non-surgical subgingival debridement, (**e**) azithromycin as adjunct systemic antibiotic, (**f**) metronidazole plus amoxicillin as adjunct systemic antibiotic, (**g**) amoxicillin as adjunct systemic antibiotic, (**h**) less of 7 days of antibiotic administration, and (**i**) more than 7 days of antibiotic administration. Weighted mean is presented at CI 95%. Heterogeneity was determined using Higgins (I^2^). A random-effects model was applied in all the analyses. Statistical significance was set at 0.05 [1,6,17,24,30,48,52,54,55,56,57].

The mean of PPD reduction, when comparisons were established between both experimental and control groups, was 0.1, ranging from −0.26 to 0.46 (CI 95%) (*p* = 0.58), indicating that the probing pocket depth is similar when antibiotics are systemically administered or not. Heterogeneity was slightly high, I^2^ = 54%, and the significance of the random-effects model was *p* = 0.58 (Figure 6a). The PPD forest plot graph is displayed in Figure 6a. Systematic heterogeneity is displayed in the funnel plot graph (Figure 6b). In order to deal with the great heterogeneity obtained, apart from the random-effects model that takes into account intra- and between-studies variability, several subgroup analyses were performed. The comparative studies performed in the four subgroups (time of follow-up, surgical therapy or not, type of antibiotics, and duration of antibiotic regimen) did not show significant differences when the control and the test groups, considering PPD, were analyzed (Figure 7).

Secondary outcomes were also determined in the present research (Table 3). Eleven papers [6,17,22,24,30,49,50,51,52,53,54] reported bone loss. A generalized reduction of approximately 0.75 mm was obtained after 12 months of follow-up (Table 3) when systemic antibiotics were administrated in conjunction with other therapies, such as mechanical debridement, bone graft plus membrane or even titanium granulates. Plaque score was also analyzed in eleven studies [1,6,16,22,47,48,50,52,54,56,57]. Plaque scores in all groups were significantly reduced from baseline to successive follow-up periods when systemic antibiotics were used in combination with complementary therapies. Suppuration was determined in eight articles [6,24,47,48,50,52,53,57], and most showed a significant reduction after systemic antibiotic administration. Eight articles [1,6,16,24,30,55,56,57] also reported total bacterial counts, and *Prevotella intermedia*/*nigrescens*, *Porphyromonas gingivalis*, and *Aggregatibacter actinomycetemcomitans* were some of the most common bacteria associated with peri-implantitis. A reduction in bacterial count was observed when other therapeutic measurements were adopted in combination with systemic antibiotics.

Four publications [16,47,50,57] tackled recession, which was significantly reduced in the majority of the papers analyzed. Three articles [6,48,57] reported information on clinical attachment level. Two papers [48,55] referred to the gingival index, and two more articles [6,53] dealt with adverse effects. Only one paper [50] reported keratinized mucosa changes (Table 3). Systemic antibiotics contributed to the results obtained in conjunction with other clinical treatments.

Non-surgical debridement plus the adjunctive use of systemic antibiotics was used in two papers [48,49], and only one used full mouth scaling and root planning [55]. Four manuscripts presented control patients treated with mechanical surface decontamination (MSD) [6,30,51,57]. Three articles showed patients treated, in the control group, with MSD in combination with photodynamic therapy (PDT) [1], surgical therapy (ST) and antiseptics [24], and probiotics [54]. The rest used non-surgical subgingival debridement and placebo [48], open flap debridement and antibiotics [52], full mouth scaling with root planning [55], mechanical debridement and photodynamic therapy [56], and bone graft plus antibiotics [17].

## 4. Discussion

### 4.1. Can Systemic Antibiotics Be Efficacious in Bleeding Reduction on Probing (BoP) and Probing Pocket Depth (PPD)?

To the best of our knowledge, this study is the first systematic review conducted to recognize the effectiveness of systemic antibiotic application in the treatment of peri-implantitis. This systematic review and meta-analysis aimed to identify the most reliable scientific information with regard to the efficacy of systemic antimicrobial administration, in terms of BoP and PPD. Following the definition proposed by Berglundh et al., 2018 [3], the definition of peri-implantitis includes: (1) bleeding and/or suppuration on gentle probing, (2) increased probing pocket depth compared to previous examinations and (3) bone loss. Thus, BoP and PPD were selected as the main outcomes of the present systematic review and meta-analysis. Regarding BoP, its diagnostic value has been widely recognized, since a strong consistency has been proven between BoP and histologically inflammatory lesions in gingival tissues [58,59]. It has also been shown to be a prognostic tool of utmost importance. Carcuac et al., 2017 [51], and Karlsson et al., 2019 [60], performed two longitudinal studies, in which the predictive value of BoP in implants was evaluated. Both came to the conclusion that while BoP had a low positive predictive value for the prediction of future bone loss, a negative result for BoP was a strong predictor for the preservation of marginal bone levels. Thus, BoP was selected as a prognostic tool due to its highly negative predictive value. However, BoP is a common diagnostic criterion with peri-implant mucositis. Hence, another primary outcome was separately evaluated and meta-analyzed in order to ease the interpretation of the results. The variation in the PPD was selected as it was included as a diagnostic factor in the last definition of peri-implantitis [3].

A thorough search of the relevant literature yielded a great variety of results interpreted as treatment for peri-implantitis. Attempting to improve homogeneity, only investigations that counted BoP and PPD reduction were included in this review. The present systematic review and meta-analysis supports that systemic antibiotic administration did not affect the results of the peri-implantitis treatment in terms of BoP (Figure 4 and Figure 5) or PPD (Figure 6 and Figure 7). The absence of clinical benefits with adjunctive systemic antibiotic therapy was also reported after treating periodontitis with these therapies in a long-term study [61].

Eighteen studies comprised the present research, of which nine were randomized clinical trials. To obtain the maximum amount of data, case series, prospective studies and case cohorts were also included. A total of 870 implants in 605 patients were analyzed, involving the maxilla and the mandible. Ten studies evaluated metronidazole, eleven assessed amoxicillin, three azithromicyn, two clindamycin, two tetracycline, and only in one study were ciprofloxacin, sulfonamide, trimetroprim, ornidazol, amoxicillin/clavulonate potassium and erythromycin employed (Table 2). Concerning the type of antibiotics systemically administered, both reduction of bleeding (BoP) and probing depth (PPD) did not vary after the antibiotic therapy (Figure 5 and Figure 7). No efficacy was shown independently of the distinct employed formulations analyzed in the subgroups of the present research (azithromycin, amoxicillin plus metronidazole, and amoxicillin). Azithromycin (Zithromax) is a macrolide antibiotic, and is a widely prescribed broad-spectrum antibacterial, particularly for respiratory infections [62]. Azithromycin is detectable in inflamed periodontal tissues beyond 14 days after systemic administration and is associated with clinical and microbiological improvement [31]. In periodontitis, in two out of four studies, this antibiotic demonstrated statistically significant benefits in PPD and BoP [63]. The rationale of using a systemic antibiotic such as metronidazole may be justified by the fact that it improves the treatment of refractory periodontitis after nonsurgical periodontal therapy [64], inhibiting the ADN synthesis. It is also normally prescribed in support of conventional periodontal therapy [65], though no clear trend has been found [63]. Notwithstanding, the reduction in implant sites with PPD > 4 mm and BoP was significantly higher in patients taking amoxicillin plus metronidazole in a post-operative regimen. However, this favorable result is not consistent across the literature [50]. Comparing the test and control groups when amoxicillin as systemic antibiotic therapy was used, in the analysis of subgroups, the mean difference was −0.30, ranging from −2.70 to 2.09, meaning that systemic amoxicillin, per se, does not promote improvements in the PPD in peri-implantitis, as the control group obtained better outcomes (Figure 7). Tetracyclines, that have also been administered in some studies reported in the present research [22,56], exhibited high substantivity to periodontal pocket hard tissues and root surfaces [66]. Erythromycin [56], in periodontal defects, contributes to bone regeneration thanks to its osteoblastic cells’ proliferation [67].

Regarding the follow-up time period, 3 months was considered as an approximated average of clinical following [6,48,57]. If two subgroups (≤3 m vs. >3 m) are considered, neither of the administered antibiotics influenced the primary outcomes considered in the present research, BoP and PPD (Figure 5 and Figure 7). In the same way, the mean difference between the experimental and control group when antibiotics were systemically administered and the follow-up was beyond 3 months was −0.07, ranging from −0.62 to 0.48, in the analysis of PPD. This indicates that after applying a follow-up longer than 3 months, the administration of antibiotics does not make sense (Figure 7).

Referring the application or not of surgical therapy, the primary outcomes did not change when antibiotics were administered in the experimental group compared with the control group (Figure 5 and Figure 7). Though systemic antibiotics did not influence BoP in the treatment of peri-implantitis, a notable results was the bleeding reduction from 100% at baseline to 7% after 6 months follow-up [30]. This reduction in BoPjustified the use of surgical interventions, even without the use of supplementary antibiotics [30]. Concerning the presence of surgical therapy in the protocols analyzed in the present research, the use of systemic antibiotics was only proved to be clinically effective when they were associated with other adjunctive clinical therapies, such as surgical [24,56] or non-surgical debridement [48,49].

Among the RCTs analyzed in the present systematic review and meta-analysis, Carcuac et al., 2016 [24], reported the highest PPD reduction (~4.8 mm) after 12 months of follow-up when antibiotics (AMX 750 mg/12 h for 10 d) were systemically administered (Table 2). It was an RCT study, comprising 51 patients/96 implants, where probing depth reduction showed the effectiveness of combining surgical therapy, mechanical surface decontamination and systemic antibiotic regimen in the regenerative therapy of peri-implantitis. In the treatment of PI disease, the systemic application of antibiotics in the test group was compared with photodynamic therapy in combination with mechanical debridement in two studies [1,56].

### 4.2. Did the Administration of Systemic Antibiotics Affect Other Secondary Outcomes That Were Analyzed?

Secondary outcomes usually become associated in peri-implantitis. Infections around titanium implants are sometimes difficult to treat due to the exposed threads and rough surface that enable plaque accumulation [6]. In our systematic review, 11 out of the 18 articles included the determined presence or absence of plaque score assessments (Table 3). The high generalized plaque scores at the beginning of the study are suggestive of the poor oral hygiene that the patients exhibited. The reduction in plaque scores from baseline to successive follow-up periods, after the administration of systemic antibiotics and other adjunctive therapies, may be due to the oral hygiene instructions and maintenance protocols performed in the study [1]. Compared with healthy implants, greater levels of titanium were detected in submucosal plaque around implants with peri-implantitis. Titanium dissolution products have been shown to alter the peri-implant microbiome structure and diversity, indicating an association between titanium dissolution products and peri-implantitis [6].

The non-linear accelerative progressive pattern of bone loss in peri-implantitis [40] leads to failure if the given infection is not proficiently arrested [50]. The bone loss is measured by the radiographic bone level, i.e., the distance between the implant shoulder or the most coronal part of the endosseous part of the implant and the bottom of the defect in bone-level implants [49]. Eleven papers, referred to in the present research, reported bone loss (Table 3). As well as by means of radiographs, to diagnose peri-implantitis, probing is a requisite. It should be noted that pus is a common finding when probing implants with peri-implantitis [68]. The absence of bleeding/suppuration on probing during follow-up after treatment of peri-implantitis has a high predictive value for no further bone loss [51]. Suppuration on probing corresponds with the presence or absence of suppuration after probing [50], and is commonly interpreted as a sign of the peri-implant osseous defects that result from peri-implantitis [6,52]. Eight papers, referred to in the present study, reported a reduction in suppuration (Table 3). Nart et al., 2020 [50], obtained a significant reduction in suppuration, from around 66% at baseline to approximately 7% after a 12-month follow-up period (Table 2 and Table 3). Jepsen et al., 2016 [52], reported significant differences in both control and test groups (from ~26 to 1% and from ~28 to 1%, respectively) after 12 months of follow-up. A similar trend was followed by Heitz Mayfield (2011) [53], who obtained a significant reduction in suppuration at 3 and 12 months after treatment (Table 3). These authors [47] also obtained a reduction in suppuration from 58% to 5.6% after 12 months of follow-up when combining the administration of systemic antibiotics with other adjunctive therapies.

Ten articles, referred to in the present systematic review, reported total bacterial counts (Table 3). A complex array of bacteria contained in dental plaque are responsible for the onset of disease and the triggering of the infection [1]. In contrast to healthy implants with a biofilm mainly composed of Gram-positive cocci, the biofilm in peri-implantitis is characterized by the predominance of Gram-negative anaerobic bacteria [57]. Leonhardt et al., 2003 [22], achieved a reduction from 73 to 36% in sites of periodontal pathogens. Bacteria in (undisturbed) biofilms, as compared to planktonic bacteria, display an increased tolerance of antimicrobial agents, which may cause adjunctive systemic antibiotics to be less effective. A reduction in *Porphyromonas gingivalis, Treponema denticola* and *Tannerella forsythia* counts at 6 months follow-up in comparison to the baseline were reported by Almohareb et al. [56]. Total bacterial load returned to initial levels after quite short time intervals of 1–2 months and increased gradually over time after nonsurgical subgingival debridement [48]; nevertheless, these authors [48] obtained a significant reduction in red complex species, by using non-surgical debridement plus metronidazole and amoxicillin, at 3 months of follow-up (Table 3). These findings are in line with the classical ecological plaque hypothesis [69], in which it is established that qualitative changes in the subgingival biofilms may lead to dysbiosis. The fact that the total bacterial load returned to initial levels after 2 months may not negatively affect the state of the patient’s health, since the red complex species were reduced after 3 months. This change in the subgingival environment may be compatible with a peri-implant healthy state. In addition, Hallström et al., 2017 [30], also reported trends of decreasing bacterial loads between the baseline and 2 and 4 weeks in both the experimental and the control groups, but without a retained reduction at later time points.

Four papers referred to gingival recession, i.e., the distance (mm) between the mucosal margin and the implant abutment interface [50] (Table 3). These measurements ranged from 4.5 mm (baseline) and 6.3 mm (after 3 m) in Heith-Mayfield (2018) [47], to −1.11 (baseline) and −2.14 (after 12 m) in Mombelli et al., 1992 [28]. Only one paper [50] reported information regarding keratinized mucosa, interpreted as the distance (mm) from the mucosal margin to the mucogingival junction, but differences between baseline and 12-month follow-up were not obtained. Most of the papers failed to evaluate the keratinized mucosa width, due to its unclear effect on peri-implant health. According to the consensus of Group 4 at the 2017 World Workshop on the Classification of Periodontal and Peri-Implant Diseases and Conditions, the evidence related to the presence or absence of keratinized mucosa as a risk/protective factor for the development of peri-implantitis is still not conclusive [3]. However, there is growing evidence that less than 2 mm of keratinized mucosa width is associated with peri-implant mucositis, which could potentially trigger future marginal loss in non-compliant patients [70,71,72]. Three articles reported measurements of clinical attachment level, i.e., the distance (mm) from the implant abutment junction to the bottom of the pocket [48]. Two papers described data concerning the gingival index [48,55] (Table 3).

Systemic antibiotics may interact with other drugs, which could lead to comorbidity, cause serious events, increase the proliferation of antimicrobial resistance and the origin of superinfections, and result in the overgrowth of opportunistic pathogens that are difficult to eradicate [73]. The risk of adverse effects should also be considered, especially when more than one antibiotic is prescribed. Local antibiotic therapy has not promoted adverse effects in the case of treatment for peri-implantitis [15]. In the present research, only two studies reported adverse effects [6,53], which were identified as headache, dizziness, diarrhea with nausea, mild gastrointestinal complaints or vaginal thrush, and which were resolved without intervention. Twenty-five percent of patients experienced adverse events related to the systemic antimicrobials following the treatment in Heitz-Mayfield (2011) [53]. It should be noted that other complementary therapies, apart from the administration of systemic antibiotics, were adopted.

### 4.3. Study Limitations and Biased Quality of the Research

The slight heterogeneity detected in the studies that report BoP values (Figure 4a), as observed in the funnel plot (Figure 4b), can be explained by the few differences in the surgical techniques implemented, the biomaterials used and the operators. On the contrary, the studies analyzing PPD, referenced in the present research, showed a higher heterogeneity (I^2^ = 54%) (Figure 6a), indicating that a random-effects model was applied [74] and meaning that more than 50% of the studies were heterogeneous. This can be considered as a study limitation that may reduce the quality of the encountered evidence. The few studies of peri-implantitis treated with systemic antibiotics that reported on long-term results were generally characterized by limited sample sizes (namely the “small studies effects” [75], as a consequence of the heterogeneity and the lack of control groups [47,76,77,78,79,80]). Thus, the understanding of the effect of different treatment protocols for advanced peri-implantitis was limited. It should be considered that the experiment’s sample size ranges from 9 to 67 patients and from 20 to 121 implants. The study with the greatest sample size (n = 67) was Carcuac et al., 2017 [51], in which an RCT study is presented, reporting, after 36 months of analysis, a PPD reduction of −3 ± 2.24 mm, and a total reduction in BoP after applying mechanical surface debridement and systemic antibiotics.

Another limitation of the present meta-analysis is the pooling together of the unit of analysis for the performance of the statistical analysis, due to the relatively small number of included studies. While some of the studies analyzed the data on a patient level, the rest used the implants as the subjects of study. This methodological limitation may have increased the type-I error [81].

The biased quality of eight of the included papers and the lack of appropriately conducted RCTs pose two more limitations of this systematic review and meta-analysis. For the present study, only nine RCTs were eligible. A meta-analysis should mainly be conducted on RCTs, which have a high level of evidence, but cases series are frequently included when RCTs are involved in a limited number. Nevertheless, the risk of bias of the included RCTs was judged as low (Figure 2), though some of them presented some concerns, such as deviation from the intended interventions and the randomization process. No RCT study showed a high risk of bias (Figure 2). On the other hand, the risk of bias in the non-RCT studies was generally considered as low, though this was unclear in some of them and a small number showed a high risk of bias, specifically with reference to the complete inclusion of participants (Figure 3). Three more factors may have influenced the clinical results: implant surface characteristics, implant location and the multiplicity of the antibiotics used. These factors might promote a different host response [15]. The follow-up of the patients included in the present review was between 10 days [16] and 54 months [49]. More well-designed RCTs are needed in order to strengthen the current evidence for systemic antibiotics and the rest of the proposed treatments in peri-implant conditions. The most recent case definitions of disease proposed in the 2017 World Workshop on the Classification of Periodontal and Peri-implant Diseases and Conditions [3] should be employed. Future studies should also employ more standardized and extended follow-up periods in order to determine the suitability of the present protocols.

## 5. Conclusions

The findings of this systematic review and meta-analysis allow us to conclude that the existing scientific evidence suggests that in patients affected by peri-implantitis, the administration of systemic antibiotics reduced neither BoP nor PPD. Nevertheless, clinicians can expect to obtain significant results in the reduction of some secondary outcomes, such as reduced clinical attachment loss, lower suppuration and recession, reduced bone loss and lower total bacterial counts, though some adverse events may also be triggered. The non-indication of systemic antibiotics in the case of peri-implantitis may contribute to manage the problem of antibiotic resistance.

## Figures and Tables

**Table 1:** Excluded studies for qualitative and quantitative synthesis with reasons.

Article	Reason for Exclusion
Cosgarea et al., 2020 [36], Hallström et al., 2012 [37], Ramos et al., 2016 [38] and Buser et al., 1990 [39]	Not treatment of peri-implantitis
Nart et al., 2018 [40], Xu et al., 2016 [41] and Tada et al., 2018 [42]	AB therapy (topical application, not as peri-implantitis treatment)
Brignardello-Petersen et al., 2017 [43]	Review
Verdugo et al., 2017 [44] and Suh et al., 2003 [45]	No PPD or BoP data
Khoury et al., 2008 [46]	Less than 3 months of follow-up
Heitz-Mayfield et al., 2018 [47]	Follow-up of included study

**Table 2:** Characteristics of the included studies. Primary outcomes investigated were BoP and PPD reduction in the treatment of peri-implantitis when using systemic antibiotics.

Author	Study Design	Patients/Implants	Control Group	Test Group	Antibiotic and Dosage	Follow-Up	BoP Mean ± SD (%)	PPD Mean ± SD (mm)
Al-Deeb 2020 [1]	RCT	30 patients 30 implants	MSD + PDT (n = 15)	MSD + AB (n = 15)	AZM 500 mg (1st day) AZM 250 mg (2–4 day)	6 w 3 m	CG: B = 12.3 ± 4.8 6 w = 7.4 ± 3.6 3 m = 8.0 ± 3.7	CG: B = 4.8 ± 1.0 6 w = 4.1 ± 1.1 3 m = 3.9 ± 0.9
							TG: B = 15.7 ± 3.9 6 w = 12.6 ± 3.8 3 m = 10.1 ± 3.1	TG: B = 4.6 ± 1.1 6 w = 4.0 ± 1.0 3 m = 3.9 ± 1.0
Shibli 2019 [48]	RCT	40 patients 40 Implants	NSD + Placebo (n = 20)	NSD + AB (n = 20)	MTZ 400 mg + AMX 500 mg 1/8 h for 14 day	3 m	CG: B = 97.0 ± 34.5 3 m = 90.0 ± 31.6	CG: B = 7.6 ± 1.8 3 m = 6.5 ± 1.9
							TG: B = 90.0 ± 31.6 3 m = 90.0 ± 31.6	TG: B = 9.9 ± 3.6 3 m = 6.9 ± 2.5
Liñares 2019 [49]	PS	18 patients 25 implants		NSD + AB (n = 25)	MTZ 250 mg 2/8 h for 7 day	54 m	NR	B = 8.72 ± 2.13 54 m = 4.06 ± 0.8
Nart 2020 [50]	PS	21 patients 21 implants		MSD + AB (n = 21)	MTZ 500 mg 1/8 h for 7 day	12 m	B = 78.78 ± 28.26 12 m = 21.22 ± 24.76	B = 5.34 ± 1.29 12 m = 3.69 ± 0.47
Carcuac 2017 * [51]	RCT	67 patients 121 implants	MSD (n = 53)	MSD + AB (n = 68)	AMX 750 mg 1/12 h	36 m	CG: B = 100 B-36 m = 0	CG: B-36 m = −2.38 ± 2.55
							TG: B = 100 B-36 m = 0	TG: B-36 m = −3.00 ± 2.24
Hallström 2017 [30]	RCT	39 patients 39 implants	OFD (n = 19)	OFD + AB (n = 20)	AZM: −250 mg × 2 the day of surgery −250 mg × 1 for 4 day	6 m	CG: B = 100 6 m = 6.3	CG: B = 5.8 ± 0.9 6 m = 4.6 ± 1.1
							TG: B = 100 6 m = 7.0	TG: B = 5.8 ± 1.0 6 m = 4.7 ± 1.3
Jepsen 2016 [52]	PS	63 patients 63 implants	OFD + AB (n = 30)	OFD + PTG + AB (n = 33)	AMX 500 mg/8 h for 8 day MTZ 400 mg/12 h for 8 day	12 m	CG: B = 85.5 ± 23.9 12 m = 40.4 ± 37.1	CG: B = 6.3 ± 1.6 12 m = 3.5 ± 1.1
							TG: B = 89.4 ± 20.7 12 m = 33.3 ± 31.7	TG: B = 6.3 ± 1.3 12 m = 3.5 ± 1.5
De Waal 2021 [6]	RCT	62 patients 143 implants	MSD (n = 68)	MSD + AB (n = 75)	AMX 500 mg/8 h 7 day MTZ 500 mg/8 h for 7 day	3 m	CG: B = 94.66 ± 9.42 3 m = 55.47 ± 31.60	CG: B = 5.82 ± 1.42 3 m = 4.42 ± 1.38
							TG: B = 85.96 ± 19.32 3 m = 47.37 ± 30.43	TG: B = 5.63 ± 1.24 3 m = 3.96 ± 1.21
Heitz-Mayfield 2012 [53]	PS	24 patients 36 implants		OFD + AB (n = 36)	AMX 500 mg/8 h for 7 day MTZ 400 mg/8 h for 7 day	3 m PPD 12 m BoP	B = 13.9 ± 11.6 3 m = NR 12 m = 6.9 ± 5.4	B = 5.3 ± 1.8 3 m = 3.0 ± 0.7
Leonhardt 2003 * [22]	PS	9 patients 26 implants		CLI (n = 5) MTZ, AMX (n = 4) Tetracycline (n = 5) MTZ, AMX (n = 3) Ciprofloxacin (n = 5) Sulfonamide, Trimetroprim (n = 2) MTZ (n = 2) Dose of antibiotics NR	CLI for 4 w MTZ, AMX for 4 w Tetracycline for 4 w MTZ, AMX for 2 w Ciprofloxacin for 2 w Sulfonamide, Trimetroprim for 2 w MTZ for 2 w	12 m	B = 100 12 m = 36	NR
Carcuac 2016 [24]	RCT	51 patients 96 implants	RT+ OFD + AS (n = 49)	RT + OFD + AB (n = 47)	AMX 750 mg/12 h for 10 day (3 day prior to surgery)	6 m	CG: B = 100 6 m = 26 ± 56.5	CG: B = 7.79 ± 1.69 B-12 m = −2.18 ± 1.54
							TG: B = 100 6 m = 16 ± 34	TG: B = 7.85 ± 1.57 B-12 m = −3.03 ± 1.58
Alqahtani 2021 [54]	RCT	28 patients 28 implants	MSD + PT (n = 14)	MSD + AB (n = 14)	AMX 500 mg/8 h for 7 day	6 m	CG: B = 48.6 ± 6.6 6 m = 20.6 ± 14.1	CG: B = 5.2 ± 0.5 6 m = 1.2 ± 0.3
							TG: B = 46.2 ± 5.4 6 m = 30.2 ± 6.4	TG: B = 5 ± 0.6 6 m = 2.6 ± 0.8
Gomi 2015 [55]	RCT	20 patients 20 implants	FM-SRP (n = 10)	FM-SRP + AB (n = 10)	AZM 500 mg/24 h for 3 day	1 w 1 m 3 m	CG: B = 25.7 ± 2.8 1 w = 18.3 ± 2.6 1 m = 17.3 ± 3.4 3 m = 19.8 ± 3.3	CG: B = 4.35 ± 0.22 1 w = 4.33 ± 1.02 1 m = 4.12 ± 0.32 3 m = 4.08 ± 0.30
							TG: B = 27.9 ± 4.3 1 w = 4.9 ± 1.8 1 m = 2.7 ± 0.4 3 m = 2.6 ± 0.4	TG: B = 4.28 ± 0.85 1 w = 3.72 ± 0.89 1 m = 3.44 ± 0.54 3 m = 3.35 ± 0.31
Mombelli 1992 * [16]	PS	9 patients		MSD + AB	Ornidazol 1.000 mg for 10 day	10 day 1 m 3 m	B = 89 10 day = 33 1 m = 89 3 m = 44	B = 5.89 10 day = 4.33 1 m = 4.33 3 m = 4.22
Almohareb 2020 [56]	RCT	40 patients 79 implants	MD +PDT (n = 43)	MD + AB (n = 36)	AMX 500 mg/8 h for 7 day MTZ 400 mg/8 h for 7 day	6 m	CG: B = 45.3 ± 14.8 6 m = 27.2 ± 13.3	CG: B = 5.2 ± 2.0 6 m = 4.4 ± 1.1
							TG: B = 43.8 ± 13.9 6 m = 29.7 ± 13.2	TG: B = 5.4 ± 2.1 6 m = 4.7 ± 1.0
Khoury 2001 [17]	PS	14 patients 21 implants	OFD + BG + AB (n = 12)	OFD + BG + RM + AB (n = 9)	4 w prior to surgery (for 1 w), and 1 day and finishing 7 day after surgery according to individual antimicrobial susceptibility test	6 m	NR	CG: B = 8.0 ± 0.5 6 m = 6.5 ± 0.8
								TG: B = 7.7 ± 0.5 6 m = 6.4 ± 0.9
Heitz-Mayfield 2016 [47]	PS	24 patients 36 implants		OFD + AB (n = 36)	AMX 500 mg/8 h for 7 day MTZ 400 mg/8 h for 7 day	12 m	B = 13.9 ± 11.6 12 m = 6.9 ± 5.4	B = 5.3 ± 1.8 12 m = 2.9 ± 0.8
Irshad 2021 [57]	PS	46 patients 46 implants	MSD (n = 21)	MSD + AB (n = 25)	AMX 500 mg/8 h for 5 day MTZ 400 mg/8 h for 5 day	3 m	CG: B = 100 3 m = 86	CG: B = 7.5 ± 1.6 3 m = 4.6 ± 1.2
							TG: B = 100 3 m = 78	TG: B = 7.6 ± 1.4 3 m = 5.2 ± 1.3

SD: standard deviation; RCT: randomized clinical trial; PS: prospective study; CG: control group; TG: test group; PDT: photodynamic therapy; AB: antibiotic; RT: resective techniques; AS: antiseptic; AZM: azithromycin; AMX: amoxicillin; MTZ: metronidazole; MSD: mechanical surface decontamination; NSD: non-surgical subgingival debridement; B: baseline; NR: not reported; OFD: open flap debridement; PTG: porous titanium granule; CLI: clindamycin; PT: probiotic; FM-SRP: full mouth-scaling and root planning; BG: bone graft; RM: resorbable membrane; MD: mechanical debridement; NR: not reported. * Studies excluded from the meta-analysis.

**Table 3:** Summary of secondary outcomes.

Author	CG/TG	Plaque Score	Gingival Index	CAL	Suppuration	Recession	Keratinized Mucosa	Bone Loss	Total Bacteria Counts	Adverse Effects
Al-Deeb 2020 [1]	CG	B:44.5 ± 9.7 6 w:15.7 ± 3.1 *	NR	NR	NR	NR	NR	NR	*P. aeruginosa* and *S. aureus* in CG and TG showed SS reductions at 12 w. On inter-group comparison, CG and TG showed no SS differences at follow-up.	NR
	TG	B: 47.4 ± 10.2 6 w: 20.1 ± 4.2 *	NR	NR	NR	NR	NR	NR		NR
Shibli 2019 [48]	CG	B: 60.0 ± 51.6 3 m: 40.0 ± 51.6	B:50.0 ± 52.7 3 m:10.0 ± 31.6	B: 7.8 ± 1.9 3 m: 6.7 ± 2.0	B: 30.0 ± 48.3 3 m: 0 *	NR	NR	NR	Both therapies led to a SS reduction in the proportion of red complex species at 3 m.	NR
	TG	B: 40.0 ± 51.6 3 m: 40.0 ± 51.6	B: 50.0 ± 52.7 3 m: 0	B: 9.9 ± 3.6 3 m: 7.1 ± 2.8 *	B: 50.0 ± 52.7 3 m: 0 *	NR	NR	NR		
Liñares 2019 [49]	TG	NR	NR	NR	NR	NR	NR	B: 4.52 ± 2.14 54 m:1.92 ± 1.93 *	NR	NR
Nart 2020 [50]	TG	B:68.17 ± 26.68 12 m:40.91 ± 29.87 *	NR	NR	B: 65.90 ± 45.57 12 m:6.82 ± 21.62 *	B: 0.17 ± 0.47 12 m:0.79 ± 0.72 *	B: 2.59 ± 1.26 12 m:1.95 ± 1.05	B: 3.76 ± 1.26 12 m:2.45 ± 1.26 *	NR	NR
Carcuac 2017 [51]	CG	NR	NR	NR	NR	NR	NR	B-36 m: 0.51 ± 1.87	NR	NR
	TG	NR	NR	NR	NR	NR	NR	B-36 m: −0.32 ± 1.35	NR	NR
Hallström 2017 [30]	CG	NR	NR	NR	NR	NR	NR	B: 4.9 ± 1.7 12 m:4.5 ± 1.5	No SS differences in changes of TBC between B and 6 or 12 m. No SS differences between groups.	NR
	TG	NR	NR	NR	NR	NR	NR	B: 4.6 ± 1.6 12 m:4.0 ± 1.6		NR
Jepsen 2016 [52]	CG	B: 21.0 ± 28.7 12 m:10.3 ± 20.0	NR	NR	B: 25.9 ± 33.1 12 m: 1.3 ± 4.6 *	NR	NR	(m) B-12 m: −0.96 ± 1.35 (d) B-12 m: −0.84 ± 1.14	NR	NR
	TG	B: 25.8 ± 36.8 12 m:24.8 ± 36.3	NR	NR	B: 27.8 ± 34.0 12 m:1.0 ± 4.2 *	NR	NR	(m) B-12 m: −3.58 ± 2.05 (d) B-12 m: −3.45 ± 2.16	NR	NR
De Waal 2021 [6]	CG	B: 42.11 ± 30.89 3 m: 6.88 ± 14.72	NR	B: 12.45 ± 2.36 3 m: 11.49 ± 2.01	B: 8.33 ± 16.67 3 m: 0	NR	NR	B: 3.03 ± 1.24 3 m:3.08 ± 1.32	No SS differences between B and 3 m, except for *T. denticola* in TG. No SS differences at 3 m.	Between groups no SS differences. In TG adverse events (headache, dizziness, diarrhea and nausea).
	TG	B: 42.35 ± 28.02 3 m: 8.20 ± 13.28	NR	B: 12.35 ± 1.68 3 m: 11.39 ± 1.62	B: 8.33 ± 16.67 3 m: 0	NR	NR	B: 2.65 ± 1.61 3 m: 2.70 ± 1.65		
Heitz-Mayfield 2012 [53]	TG	NR	NR	NR	Highly significant reduction in suppuration at 3 m maintained until 12 m.	NR	NR	3 implants in 3 patients gained bone, the rest had stable crestal bone levels.	NR	Six patients reported mild adverse effects: gastrointestinal (5) or vaginal thrush (1).
Leonhardt 2003 [22]	TG	B: 100 12 m: 8%	NR	NR	NR	NR	NR	B: 0 12 m: 12%	B: 73% 12 m: 36%	NR
Carcuac 2016 [24]	CG	NR	NR	NR	B: 33 ± 67.3 6 m: 9 ± 19.6	NR	NR	B-12 m: −0.69 ± 1.32 *	SS decline during the 12 m period for both groups. No differences between groups.	NR
	TG	NR	NR	NR	B: 34 ± 72.2 6 m: 5 ± 10.6	NR	NR	B-12 m: 0.18 ± 1.15 *		NR
Alqahtani 2021 [54]	CG	Significantly higher at B compared with 3 and 6 m.	NR	NR	NR	NR	NR	No SS difference in m and d CBL in all groups up to 6 m.	NR	NR
	TG		NR	NR	NR	NR	NR		NR	NR
Gomi 2015 [55]	CG	NR	The GI improved in both groups, being more pronounced in the TG.	NR	NR	NR	NR	NR	In the CG, the TCB did not change over time. In TG, the TCB seemed to be clearly reduced compared with the CG.	NR
	TG	NR		NR	NR	NR	NR	NR		NR
Mombelli 1992 [16]	TG	B: 0.56 12 m: 0.86	NR	NR	NR	B: −1.11 12 m: −2.14	NR	NR	The flora was drastically reduced after therapy. At 12 m the organisms re-emerged in several treated sites.	NR
Almohareb 2020 [56]	CG	B: 38.6 ± 9.5 6 m: 21.8 ± 9.1 *	NR	NR	NR	NR	NR	NR	SS differences were observed in values for Pg, Td, and Tf at 6 m in comparison to B for both groups.	NR
	TG	B: 41.2 ± 11.7 6 m: 20.1 ± 7.7 *	NR	NR	NR	NR	NR	NR		NR
Khoury 2001 [17]	CG	NR	NR	NR	NR	NR	NR	B: 7.3 ± 1.3 6 m: 6.9 ± 1.1	NR	NR
	TG	NR	NR	NR	NR	NR	NR	B:7.4 ± 0.9 6 m: 7.0 ± 1.3	NR	NR
Heitz-Mayfield 2016 [47]	TG	B: 16.8 ± 12.7 12 m: 11.1 ± 9.2	NR	NR	B: 21 ± 58 12 m: 2 ± 5.6 *	B: −12 m: 1.0 ± 0.9	NR	NR	NR	NR
Irshad 2021 [57]	CG	B: 36 3 m: 38	NR	B: 12.0 ± 1.8 3 m:10.4 ± 1.6 *	B: 27 3 m:8 *	B: 4.5 ± 2.0 3 m: 6.3 ± 1.6 *	NR	NR	Differences between the TCB of the two groups at B were not significant.	NR
	TG	B: 30 3 m: 10 *	NR	B: 11.0 ± 1.7 3 m: 10.6 ± 1.7	B: 19 3 m: 8	B: 3.8 ± 1.4 3 m: 4.5 ± 2.3 * ^a^	NR	NR		NR

CG: control group; TG: test group; CAL: clinical attachment level; B: baseline; NR: not reported; SS: statistically significant; m: mesial; d: distal. * Statistically significant difference between follow-up period and baseline. ^a^ Statistically significant difference between groups.

## Data Availability

The data presented in this study are available on request from the corresponding author.
